# Blood glucose trajectories and incidence of diabetes mellitus in Ugandan people living with HIV initiated on dolutegravir

**DOI:** 10.1186/s12981-023-00510-6

**Published:** 2023-03-13

**Authors:** Frank Mulindwa, Barbara Castelnuovo, Nele Brusselaers, Robert Bollinger, Joshua Rhein, Mutebi Edrisa, Allan Buzibye, Willington Amutuhaire, George Yendewa, Sarah Nabaggala, Eva Laker Agnes Odongpiny, Ronald Kiguba, Aisha Nakawooza, Simon Dujanga, Martin Nabwana, Jean-Marc Schwarz

**Affiliations:** 1 Capacity Building Program, Makerere University Infectious Diseases Institute, Kampala, Uganda; 2 Global Health Institute, Antwerp University, Antwerp, Belgium; 3 Centre for Translational Microbiome Research, Department of Microbiology, Tumour and Cell Biology, Karolinska University, Stockholm, Sweden; 4 School of Medicine, Johns Hopkins University, Baltimore, USA; 5 School of Medicine, Division of Infectious Diseases, University of Minnesota, Minneapolis, MN USA; 6 Internal Medicine Department, Makerere University, College of Health Sciences, Kampala, Uganda; 7 Department of Internal Medicine, Case Western Reserve University, Cleveland, USA; 8 Department of Pharmacology and Therapeutics, College of Health Sciences Makerere University, Kampala, Uganda; 9 Makerere University Johns Hopkins Collaboration HIV Clinic, Kampala, Uganda; 10 School of Medicine, University of California San Francisco, San Francisco, USA; 11 Department of Basic Sciences, Touro University California College of Osteopathic Medicine, Vallejo, CA USA

**Keywords:** HIV, Type 2 diabetes mellitus, Dolutegravir, HIV, Incidence, Integrase inhibitors

## Abstract

**Background:**

Following reports of anti-retroviral therapy (ART) experienced Ugandan people living with HIV (PLHIV) presenting with diabetic ketoacidosis weeks to months following a switch to dolutegravir (DTG), the Uganda Ministry of Health recommended withholding DTG in both ART naïve and experienced PLHIV with diabetes mellitus (T2DM), as well as 3-monthly blood glucose monitoring for patients with T2DM risk factors. We sought to determine if the risk of T2DM is indeed heightened in nondiabetic ART naïve Ugandan PLHIV over the first 48 weeks on DTG.

**Methods:**

Between January and October 2021, 243 PLHIV without T2DM were initiated on DTG based ART for 48 weeks. Two-hour oral glucose tolerance tests (2-h OGTT) were performed at baseline, 12, and 36 weeks; fasting blood glucose (FBG) was measured at 24 and 48 weeks. The primary outcome was the incidence of T2DM. Secondary outcomes included: incidence of pre-Diabetes Mellitus (pre-DM), median change in FBG from baseline to week 48 and 2-h blood glucose (2hBG) from baseline to week 36. Linear regression models were used to determine adjusted differences in FBG and 2hBG from baseline to weeks 48 and 36 respectively.

**Results:**

The incidence of T2DM was 4 cases per 1000 PY (1/243) and pre-DM, 240 cases per 1000 person years (PY) (54/243). There was a significant increase in FBG from baseline to week 48 [median change from baseline (FBG): 3.6 mg/dl, interquartile range (IQR): − 3.6, 7.2, *p*-value (*p*) = 0.005] and significant reduction in 2hBG (2hBG: − 7.26 mg/dl, IQR: − 21.6, 14.4, *p* = 0.024) at week 36. A high CD4 count and increased waist circumference were associated with 2hBG increase at week 36.

**Conclusion:**

We demonstrated a low incidence of T2DM in Ugandan ART-naïve patients receiving DTG. We also demonstrated that longitudinal changes in BG were independent of conventional risk factors of T2DM in the first 48 weeks of therapy. Restricting the use of dolutegravir in Ugandan ART naïve patients perceived to be high risk for diabetes mellitus may be unwarranted.

**Supplementary Information:**

The online version contains supplementary material available at 10.1186/s12981-023-00510-6.

## Background

In the early anti-retroviral therapy (ART) era, nucleoside reverse transcriptase inhibitors (NRTIs) including didanosine, zidovudine, stavudine, or lamivudine were coupled with non-nucleoside reverse transcriptase inhibitors (NNRTIs) [1, 2]. Some of these combinations were linked to metabolic complications including metabolic syndrome, lipodystrophy and diabetes mellitus (T2DM) [3–5]. Since then, anti-retroviral therapy (ART) has typically included NNRTIs, protease inhibitors (PIs) and lately the preferred integrase strand transfer inhibitors (INSTIs) as anchor drugs coupled with largely metabolically safe NRTIs like tenofovir [6, 7].

After multiple countries reported primary resistance to NNRTIs above the recommended 10% threshold, the World Health Organisation (WHO) recommended the use of dolutegravir (DTG), a later generation integrase inhibitor as part of first and second line therapy [8, 9]. Multiple prospective studies reported a very good safety profile of DTG leading to high tolerability, enhanced efficacy and a high genetic barrier to resistance [10–13]. Since then, multiple countries have adopted DTG-based ART as first line therapy in programmatic settings, especially in sub-Saharan Africa where the burden of HIV is highest [14, 15].

Uganda adopted DTG-based ART use in 2018, pioneered by selected centres including the Makerere University Infectious Diseases Institute (IDI). Patients were actively switched from NNRTIs and PIs to DTG-based ART [16]. In the first year of use, the IDI reported sixteen cases of patients presenting with diabetic ketoacidosis (DKA) a few weeks to months after being switched to DTG preceded by weight loss, a phenotypical sign of insulin deficiency [17]. Fifteen of the patients were ART exposed and one, ART naïve. One shortcoming of the report was that the patients’ diabetes status was unknown at the time of switch. Following that and other anecdotal field reports, the Uganda Ministry of Health (MoH) recommended avoiding the use of DTG in Ugandan ART naïve and experienced PLHIV known to have diabetes mellitus and withholding DTG in patients who develop type-2 diabetes mellitus (T2DM). Additionally, the guidelines encouraged three monthly blood glucose (BG) monitoring for patients with one or more of these factors; age ≥ 45 years, BMI ≥ 24 kg/m^2^ and history of hypertension at baseline [18].

Multiple similar case reports of accelerated hyperglycaemia have been published [19–24]. Large population cohort studies have however given conflicting reports about the association between integrase inhibitor use and incident diabetes mellitus [25–27]. It is still unclear whether patients who present with accelerated hyperglycaemia post-initiation of DTG-based ART have a special predisposition to developing T2DM, or if DTG truly increases risk of incident T2DM at population level [28]. In the Ugandan setting, where resources are limited, the adoption of these restrictive guidelines to the use of DTG has implications. There may be missed opportunities in starting certain patient groups on DTG as well as possible over-screening for diabetes in populations perceived to have an increased risk for DM.

The reported cases of accelerated hyperglycemia that informed the Ugandan MoH guidelines were largely ART experienced patients whose diabetes status was unknown at the time of the switch to DTG. From that evidence, the Uganda MoH guidelines that followed thereafter on the use of DTG were restrictive to both ART naïve and experienced PLHIV. We set out to determine if the risk of diabetes is indeed heightened in nondiabetic Ugandan ART naïve patients in the first 48 weeks of DTG based ART. We also assessed fasting and 2-h oral glucose tolerance test (OGTT) blood glucose time course changes over 48 weeks.

## Methods

### Study design and setting

The GLUMED (Glucose metabolism changes in Ugandan PLHIV on Dolutegravir) study was a prospective cohort study at the Kisenyi Health Center IV HIV clinic in Uganda’s capital city, Kampala. This clinic has a total of 12,000 active PLHIV in care and the HIV program is supported by the Makerere University Infectious Diseases Institute with funding from the Center for Disease Control (CDC) and the U.S. President's Emergency Plan for AIDS Relief (PEPFAR).

### Study participants and study processes

Between January and October 2021, ART naïve PLHIV ≥ 18 years starting DTG based ART were screened for study inclusion. Pregnant women and very sick patients deemed unable to undergo a 2 h–75 g oral glucose tolerance test (2 h-OGTT) were excluded. Criteria for further exclusion during follow up included: new pregnancy and poor adherence to ART (adherence < 85% determined by pill count and self-reporting [18]). Patients with poor adherence were excluded to ascertain exposure to DTG based ART.

After consenting, patients were scheduled for review in 24–48 h after an overnight fast of 8–12 h. Baseline demographic, clinical and social data were collected which included: age, sex, CD4 count, body mass index (BMI), level of education, area of residence, blood pressure, waist circumference, tuberculosis status, smoking status, physical activity measured by the Global Physical Activity Questionnaire (GPAQ), alcohol consumption measured by the Alcohol Use Disorders Identification Test (AUDIT), serum creatine and serum lipid profiles. Fasting blood glucose (FBG) was measured after which patients were given an oral solution containing 75 g of glucose to be taken within five minutes. Blood glucose was measured at 30, 60, 90 and 120 min from the time of ingestion of the glucose solution using ACCU-CHECK™ glucometers from Roche diagnostics [29]. Patients found to have a normal 2 h-OGTT (FBG < 126 mg/dl and 2-h blood glucose (2hBG) < 200 mg/dl) were enrolled for 48-week follow up on tenofovir/lamivudine/dolutegravir (TDF/3TC/DTG) as recommended by the Uganda National HIV treatment guidelines [18]. Enrolled patients received the same adherence and positive living counselling package as the other patients in the Kisenyi HIV clinic before ART initiation.

Enrolled patients were prospectively followed up with BMI, waist circumference, adherence counselling, assessment of concurrent medications and clinical assessments at 12, 24, 36 and 48 weeks. Repeat 2 h-OGTT was performed at 12 and 36 weeks while FBG was measured at 24 and 48 weeks. ART adherence was evaluated on every clinical visit using self-reports and pill counts as recommended by the Uganda MoH guidelines [30].

### Outcomes

The primary outcome of the study was incidence of T2DM. Secondary outcomes were: incidence of pre-DM and median changes in FBG from baseline to 48 weeks and 2hBG from baseline to 36 weeks. T2DM was defined as a FBG ≥ 126 mg/dl or 2hBG ≥ 200 mg/dl. Pre-DM was defined as a FBG of 100 mg–125 mg/dl or 2hBG between 140 and 199 mg/dl [31].

### Statistical analysis

Data were collected by a clinical team including a study doctor, nurse and lab technician. Double entry of data was performed with external data quality assurance provided by the IDI study monitoring team. Data were exported for statistical analyses. CD4 cell count, age, serum creatine, serum lipids, changes in BMI and BG were reported as continuous variables and the rest of the variables as categorical data. BMI was categorized according to the WHO into: underweight (< 18.5 kg/m^2^), normal weight (18.5–24.9 kg/m^2^), overweight (25.0–29.9 kg/m^2^) and obesity (≥ 30 kg/m^2^) [32]. Waist circumference was categorized according to the WHO cut offs; Normal (≤ 94 cm) [Men (M)]; ≤ 80 cm [Women (W)], Increased risk of cardiometabolic complications [95–102 cm (M); 81–88 cm (W)] and substantially increased risk of cardiometabolic complications [> 102 cm (M); > 88 cm (W)] [33]. Blood pressure was categorized as: normal (< 120/80 mmHG), pre-hypertension (120–139/80–89 mmHG) and hypertension (≥ 140/90 mmHG) according to the Joint National Committee 8 (JNC-8) guidelines [34]. Participants were staged into HIV clinical stages 1, 2, 3 and 4 according to the WHO [35]. Physical activity was reported as meeting WHO recommendations on physical activity (≥ 600 Metabolic Equivalent of Task (MET) minutes per week) or not meeting WHO recommendations on physical activity (< 600 MET minutes per week) [36]. Virologic suppression was categorized according to the Uganda HIV treatment guidelines into suppressed viral load (VL) < 1000 copies/ml and non-suppressed (VL ≥ 1000 copies/ml) [30]. Laboratory normal ranges for serum creatine, fasting low density lipoproteins (LDL), high density lipoproteins (HDL) and total cholesterol (TC) were 0.72–1.24 mg/dl, 0–127.6 mg/dl, 34.8–56.07 mg/dl and 0–200 mg/dl respectively [37].

We compared characteristics between study participants who completed follow-up to those who did not. Categorical data were presented as proportions while continuous variables were presented as medians with their corresponding interquartile ranges (IQR). Post-baseline FBG and 2hBG was compared with the baseline using Wilcoxon signed rank test.

Multiple linear regression models were used to determine adjusted differences in blood glucose change between baseline and week 48 for FBG or baseline and week 36 for the 2hBG. Variables that had *p*-values < 0.1 or those with known biological plausibility and clinical significance were included in the multivariable model. Baseline BG was adjusted for in both models. Statistically significant differences were tested at a *p*-value of less than 0.05 and all *p*-values were two-sided. All analyses were done using Stata Release 17.0.

## Results

**Fig. 1:**
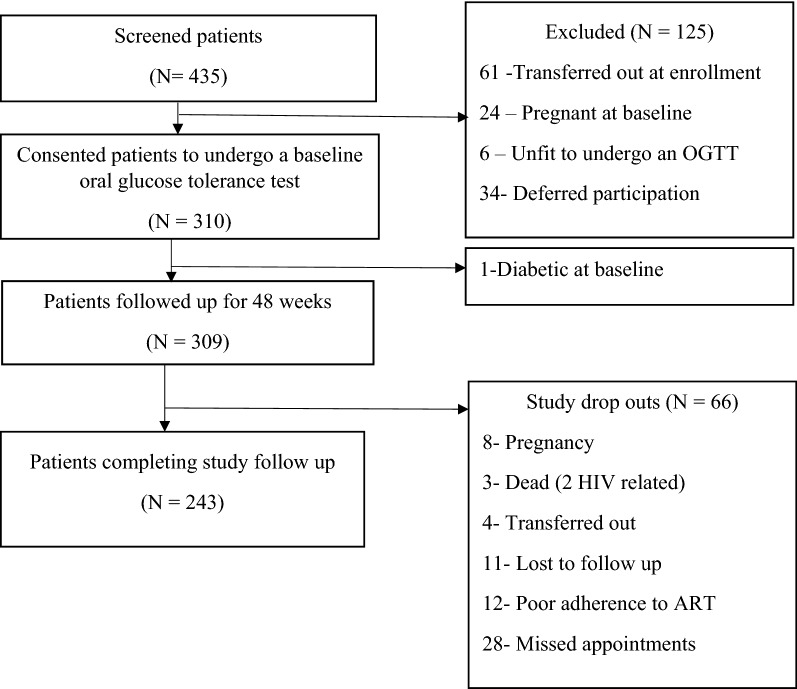
Study participant enrollment schema

Out of the 435 patients screened for enrollment, 309 patients (71.0%) were enrolled with 243 patients completing 48 weeks of follow up (21.7% drop-out) (Fig. 1).

### Baseline demographic and clinical characteristics of the study participants

Of the 243 patients enrolled, 140 (58%) were female. The median age of the participants was 31 years (IQR:27–38) with a median CD4 cell count of 318 cells/mm^3^ (IQR 163–524). Majority [N = 192 (79%)] of the patients were in HIV clinical stage 1 with 35 (14%), 14 (5.8%), 2 (0.8%) being in HIV clinical stages 2, 3 and 4 respectively. Nine (3.7%) patients had an established diagnosis of active tuberculosis diagnosed at enrollment into HIV care. Overall, 61% had a normal BMI, 23.5% were overweight, 10% underweight and 5.8% obese. Most (81%) of the patients self-reported as meeting WHO recommendations on physical activity for health. The median serum creatine, fasting LDL, fasting HDL and fasting total cholesterol at baseline were: 0.83 mg/dl, 78.1 mg/dl, 31.7 mg/dl and 136.1 mg/dl respectively. At 24 weeks of follow up, 238 patients (99.6%) had viral suppression (Table 1).

**Table 1:** Baseline clinical and demographic characteristics of the study population

Characteristic	Total (%)
Age (years), median (IQR)	31 (27, 38)
Sex	
Female	140 (57.6)
Male	103 (42.4)
Baseline CD4 cell count (cells/mm^3^), median (IQR)	318 (163, 524)
Level of education	
Uneducated	3 (1.2)
Primary	129 (53.1)
Secondary	99 (40.7)
Tertiary	12 (4.9)
Religion	
Christian	188 (77.4)
Muslim	55 (22.6)
Residence	
Rural	18 (7.4)
Urban	225 (92.6)
Employment	
No	34 (14)
Yes	209 (86)
Marital status	
Single	135 (55.6)
Married	108 (44.4)
Tuberculosis status	
No symptoms	203 (83.5)
TB suspect	31 (12.8)
TB disease	9 (3.7)
Baseline blood pressure (mmHG)	
Normal BP	175 (72)
Pre-hypertension	48 (19.8)
Hypertension	20 (8.2)
HIV clinical stage	
Stage 1	192 (79)
Stage 2	35 (14.4)
Stage 3	14 (5.8)
Stage 4	2 (0.8)
Body Mass Index (BMI) (kg/m^2^)	
Underweight (< 18.5)	24 (9.9)
Normal (18.5–24.9)	148 (60.9)
Overweight (25.0–29.9)	57 (23.5)
Obese (≥ 30)	14 (5.8)
Waist circumference (cm)	
Normal	160 (65.8)
Increased risk of cardiometabolic complications	45 (18.5)
Substantially increased risk of cardiometabolic complications	38 (15.6)
Smoking status	
Smoker	14 (5.8)
Non-smoker	229 (94.2)
Physical activity (MET minutes)	
GPAQ < 600 MET minutes	46 (18.9)
GPAQ ≥ 600 MET minutes	197 (81.1)
Alcohol consumption	
No consumption	139 (57.2)
Low risk alcohol consumption	67 (27.6)
Hazardous alcohol consumption	19 (7.8)
Risk of alcohol dependence	18 (7.4)
24-week viral loads (copies/ml) (Proxy baseline VL)	
Virologically suppressed	238 (99.6)
Unsuppressed Viral load	1 (0.4)
Change in BMI from baseline to week 48, median (IQR)	1.1 (0, 2.3)
Laboratory investigations, median (IQR)	
Creatinine (mg/dl)	0.83 (0.72, 0.95)
LDL (mg/dl)	78.1 (59.6, 94.0)
HDL (mg/dl)	31.7 (25.9, 39.8)
Total cholesterol (mg/dl)	136.1 (117.9, 158.6)
Triglycerides (mg/dl)	90.4 (69.1, 117.8)

MET, metabolic equivalent of task; IQR, interquartile range; LDL, low density lipoproteins; HDL, high density lipoproteins; GPAQ, global physical activity questionnaire; VL, viral load; BMI, body mass index

There were not significant baseline study characteristic differences between patients who completed 48 weeks of follow up and those who did not (Additional file 1: Table S1).

### Incidence of pre- diabetes and diabetes Mellitus

The incidence of DM was 4 cases per 1000 PY (1/243, diagnosed at 36 weeks). The incidence of pre-DM was 240 cases per 1000 person-years (PY) (54/243). Of the 54 patients diagnosed with pre-DM, 40 patients had a subsequent visit after pre-DM diagnosis (diagnosed between 12 and 36 weeks) and majority 32/40 (80%) reverted to a normal blood glucose state within the next 12 weeks.

### Fasting and 2-h OGTT blood glucose trajectories over 48 weeks

**Fig. 2:**
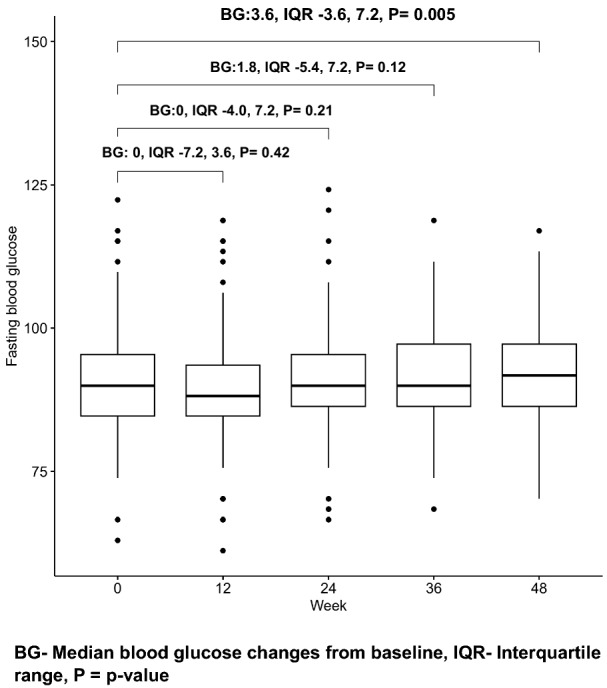
Box plots of changes in median fasting blood glucose over 48 weeks

There was an insignificant reduction in median FBG at 12 weeks followed by a steady rise through 24 weeks, 36 weeks to 48 weeks. The median FBG at 48 weeks was significantly higher than that at baseline (FBG: 3.6, IQR: − 3.6, 7.2, *p* = 0.005) (Fig. 2).

**Fig. 3:**
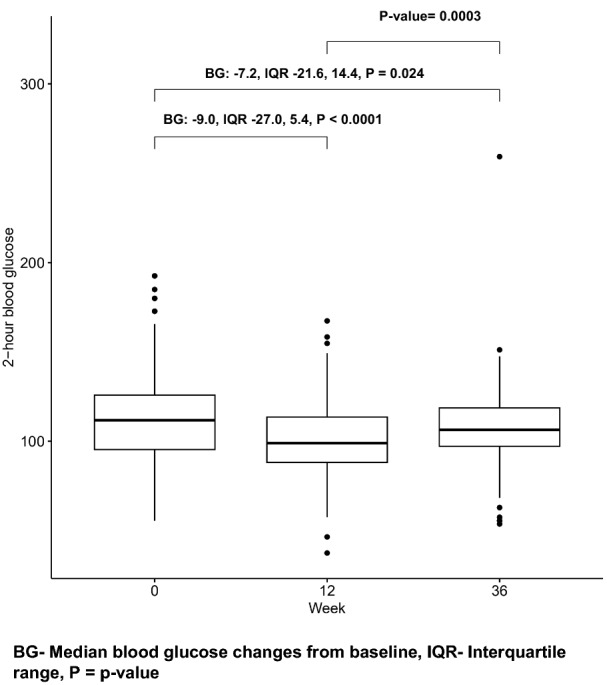
Box plots of changes in median 2-h OGTT blood glucose over 48 weeks

There was a significant decrease in the median 2hBG at 12 weeks (2hBG: − 9.0, IQR − 27.0, 5.4, *p* < 0.0001). Thereafter, was a slight increase to 36 weeks. Despite the rise in 2hBG from week 12 to week 36, the median 2hBG at 36 weeks was significantly lower than at baseline (2hBG: − 7.2, IQR: − 21.6, 14.4, *p* = 0.024) (Fig. 3).

### Factors associated with changes in blood glucose

A higher baseline CD4 cell count was significantly associated with increase in 2hBG at week 36 [adjusted median change in 2hBG: 0.01, 95% confidence interval (CI): 0.0002–0.021, *p*-value (*p*) = 0.045]. Additionally, having a waist circumference corresponding to increased risk of cardiometabolic complications at baseline was associated with an increase in 2hBG at week 36 (adjusted median change in 2hBG: 12.85, 95% CI 4.80–20.89, *p* = 0.002). There were no factors associated with increase in FBG at 48 weeks (Table 2).

**Table 2:** Factors associated with changes in fasting and 2-h blood glucose over the follow up period

	Fasting blood glucose (0–48 weeks)				2-h blood glucose (0–36 weeks)			
Characteristic^*^	Crude change (95% CI)	*p*-value	Adjusted change (95% CI)	*p*-value	Crude change (95% CI)	*p*-value	Adjusted change (95% CI)	*p*-value
Age								
	− 0.11 (− 0.25, 0.04)	0.152	0.6 (− 0.10, 0.22)	0.486	− 0.08 (− 0.61, 0.46)	0.776	0.08 (− 0.32, 0.49)	0.680
Sex								
Female	Ref.		Ref.		Ref.		Ref.	
Male	− 2.59 (− 5.06, − 0.12)	**0.040**	− 2.24 (− 4.98, 0.50)	0.108	− 3.60 (− 10.73, 3.53)	0.321	3.25 (− 4.20, 10.70)	0.391
Baseline CD4 cell count								
	0.0001 (− 0.005, 0.006)	0.970	0.002 (− 0.005, 0.005)	0.943	0.006 (− 0.006, 0.019)	0.313	0.01 (0.0002, 0.021)	**0.045**
Tuberculosis status								
No symptoms	Ref.				Ref.			
TB suspect	2.07 (− 1.82, 5.97)	0.295			− 4.93 (− 17.02, 7.16)	0.423		
TB disease	0.55 (− 6.11, 7.22)	0.870			1.24 (− 18.29, 20.76)	0.901		
Baseline blood pressure								
Normal BP	Ref.		Ref.		Ref.		Ref.	
Pre-hypertension	− 2.98 (− 5.69, − 0.27)	**0.031**	− 0.89 (− 3.62, 1.83)	0.519	0.19 (− 9.96, 10.34)	0.970	− 3.15 (− 10.72, 4.41)	0.412
Hypertension	− 2.55 (− 7.11, 2.00)	0.270	0.99 (− 4.30, 6.28)	0.712	− 5.04 (− 18.88, 8.80)	0.474	4.00 (− 8.15, 16.14)	0.517
HIV clinical stage								
Stage 1	Ref.				Ref.		Ref.	
Stage ≥ 2	1.49 (− 1.67, 2.876)	0.238	2.67 (− 2.11, 7.82)	0.262	− 18.00 (− 23.87, 3.210)	0.312	− 2.24 (− 13.01, 7.432)	0.317
Body Mass Index (BMI)								
Underweight (< 18.5)	Ref.		Ref.		Ref.		Ref.	
Normal (18.5–24.9)	0.07 (− 4.54, 4.68)	0.977	2.25 (− 1.98, 6.48)	0.295	− 11.00 (− 25.58, 3.58)	0.139	− 3.18 (− 14.75, 8.39)	0.588
Overweight (25.0–29.9)	− 2.94 (− 7.76, 1.89)	0.232	− 2.05 (− 7.32, 3.22)	0.444	− 7.62 (− 23.53, 8.29)	0.346	− 8.07 (− 21.36, 5.23)	0.233
Obese (≥ 30)	− 2.88 (− 10.00, 4.23)	0.426	− 1.77 (− 8.97, 5.43)	0.628	− 10.97 (− 27.08, 5.14)	0.181	− 12.91 (− 28.08, 2.26)	0.095
Waist circumference								
Normal	Ref.		Ref.		Ref.		Ref.	
Increased risk of cardiometabolic complications	− 0.49 (− 3.36, 2.39)	0.738	1.81 (− 1.79, 5.42)	0.322	5.18 (− 4.46, 14.82)	0.291	12.85 (4.80, 20.89)	**0.002**
Substantially increased risk of cardiometabolic complications	− 1.95 (− 5.47, 1.58)	0.277	3.56 (− 0.50, 7.62)	0.086	2.50 (− 6.35, 11.34)	0.579	13.79 (4.14, 23.45)	**0.005**
Smoking status								
Non-smoker	Ref.				Ref.			
Smoker	0.55 (− 5.32, 6.42)	0.853			10.722 (− 4.56, 26.00)	0.168		
Physical activity								
GPAQ < 600 MET minutes	Ref.		Ref.		Ref.		Ref.	
GPAQ ≥ 600 MET minutes	− 1.25 (− 4.46, 1.96)	0.444	− 2.72 (− 5.60, 0.16)	0.064	3.75 (− 4.99, 12.48)	0.399	− 2.87 (− 10.50, 4.76)	0.459
Alcohol consumption								
No consumption	Ref.				Ref.			
Low risk alcohol consumption	− 1.32 (− 4.12, 1.48)	0.353			0.86 (− 6.70, 8.42)	0.823		
Hazardous alcohol consumption	− 1.65 (− 5.54, 2.24)	0.405			14.93 (2.88, 26.99)	**0.015**		
Risk of alcohol dependence	4.23 (− 2.12, 10.58)	0.191			3.48 (− 14.49, 21.46)	0.703		
Change in BMI from baseline to Week 48								
	0.01 (− 0.61, 0.62)	0.985	0.11 (− 0.43, 0.64)	0.698	− 0.95 (− 3.19, 1.29)	0.405	1.08 (− 0.71, 2.88)	0.235
Laboratory investigations								
Creatinine (mg/dl)	− 8.59 (− 14.71, − 2.46)	**0.006**	− 2.04 (− 10.63, 6.55)	0.698	− 12.10 (− 26.33, 2.12)	0.095	− 9.19 (− 22.3, 3.93)	0.169
LDL (mg/dl)	− 0.02 (− 0.07, 0.03)	0.470			− 0.03 (− 0.17, 0.11)	0.679		
HDL (mg/dl)	0.03 (− 0.05, 0.11)	0.439			0.10 (− 0.16, 0.36)	0.467		
Total cholesterol (mg/dl)	− 0.004 (− 0.04, 0.04)	0.851			0.04 (− 0.08, 0.16)	0.510		
Triglycerides (mg/dl)	− 0.003 (− 0.03, 0.02)	0.807			0.09 (0.03, 0.16)	**0.005**	0.06 (− 0.0005, 0.112)	0.052

Bold values indicate statistically significant *p* values (*p* < 0.05)

CD4, cluster of differentiation 4; BP, blood pressure; BMI, Body Mass Index; GPAQ, Global Physical Activity Questionnaire; HDL, high density lipoproteins; LDL, low density lipoprotein

^*^Variables that had *p*-values < 0.1 or those with known biological plausibility and clinical significance were included in the multivariable model. Baseline blood glucose was adjusted for in both models

## Discussion

In this study, we determined that over 48 weeks of DTG-based ART, the incidence of T2DM was low [4 cases per 1000 PY (0.4%)] with the majority of the patients diagnosed with pre-DM having transient increase in FBG/2hBG.

The incidence rate reported in our study is similar to the rates reported in the safety data of two DTG efficacy randomized controlled trials (RCTs) with ART naïve PLHIV in Cameroon and South Africa. The ADVANCE (96-week follow up) and NAMSAL (48-week follow up) studies reported incidences of 0.9% and 0.3% with controls on NNRTIs having 0.3% and 0% respectively [38, 39]. Patients from these two studies had mean ages of 33 and 36 years respectively slightly above the 31 years in our study. Other DTG efficacy trial safety data with ART naïve patients from Europe and North America reported similar rates of incident diabetes. The FLAMINGO and SINGLE trials reported incidence rates of 0.4% and 0.5% with controls having 0.8% and 0.2% respectively over 48 weeks of follow up [40, 41]. These RCTs had primarily virologic outcomes with BG reported in safety data using Division of AIDS grading of hyperglycemia. In two large population cohort studies with PLHIV from North America and median follow up time of 24–36 months, the incidence of diabetes in patients on DTG was 2% and 1.7% compared to 3.6% in patients on NNRTI and 1% in patients on PIs, slightly above what we determined in our study population [25, 26]. Of note, these two studies were retrospective database cohorts with different criteria used for T2DM diagnosis including: HBA1C, fasting blood glucose, random blood glucose and being on diabetes drugs versus our study which was a prospective study with a strict criterion for T2DM diagnosis.

From the Makerere University Infectious Diseases Institute report, over 12 months the reported incidence of T2DM was 4·7 per 1000 PY in the case (ART naïve and exposed PLHIV started on DTG) versus 0·32 per 1000 PY in the control group (PLHIV on other first line regimens without DTG). Almost all (15/16) of the T2DM cases were ART exposed before the switch to DTG. One limitation of that report was that the diabetes statuses of patients were not known at the time of ART switch and the cases reported were the overtly symptomatic DKA patients hence a possible underestimation of the burden of hyperglycemia. The possible underestimation of the burden of incident T2DM may partially have informed the MoH guidelines. In contrast, our study recruited only patients free of T2DM at baseline with prospective proactive screening for T2DM irrespective of symptoms using a strict criterion for T2DM diagnosis hence may better represent the incidence estimates in the naïve group, which is low.

In our study, there was a drop in both FBG and 2hBG in the first 12 weeks of follow-up, followed by a consistent increase up to 48 weeks. Despite the 48-week FBG being significantly higher than at baseline, the 2hBG at 36 weeks was less. The trajectories suggested a steady increase in BG after 12 weeks suggesting that with longer follow up there could be a consistent significant increase in both fasting and 2hBG. Multiple factors interact in regulating blood glucose. These include: basal insulin secretion, post prandial insulin secretion, hepatic gluconeogenesis, insulin resistance and insulin clearance [42–44]. Systemic inflammation is known to impair insulin signaling at end organs leading to insulin resistance as well as insulin production. There is evidence documenting reduction in systemic inflammation when ART is introduced in PLHIV [45, 46]. Some studies have even suggested the reduced inflammation is even more evident in patients on integrase inhibitors compared to other ART anchors drugs [47, 48]. The initial improvement in 2hBG may be explained by an initial reduction in systemic inflammation in the first 3 months of therapy. With stabilization on ART, patients get a ‘return to health phenomenon’ where their appetite, weight and functionality improve, all factors known to impair insulin sensitivity in the long term. This could explain the subsequent rising trend in BG after week 12.

From our analysis, both FBG and 2hBG changes between baseline to 48 weeks were not associated to conventional risk factors for diabetes including: baseline BMI, physical inactivity and increase in BMI over the follow up period, apart from increased waist circumference which was associated with increase in 2hBG at 36 weeks. In the Ugandan HIV treatment guidelines, these are the parameters used to stratify T2DM risk to determine which patients to screen for T2DM every 3 months [49]. Additionally, patients tended to have improvement in glucose measurements in the first 3–6 months, the same period recommended for intensified monitoring for patients with risk factors to incident T2DM.

Our study had various limitations. We did not have a comparator group hence we could only describe the natural history of glucose changes in our cohort but not conclusively attribute the changes in BG to exposure to dolutegravir. It was a single center study in an urban area hence the results may not be generalizable to the general PLHIV population in Uganda. Much as the patients that were excluded during the study did not have significantly different clinical and demographic characteristics compared to those that completed the study, the dropout rate was high which could lead to under- or over-estimation of our end point depending on the outcome of the dropout participants. At enrollment, very sick patients were excluded, the same group of patients that may have more heightened systemic inflammation with different glucose metabolism trends on introduction of ART. Despite the limitations, our study had a clearly defined metabolic end point with a strict and sensitive criterion for the diagnosis of T2DM making our results reliable [50]. We screened patients for factors known to compound glucose metabolism and evaluated if these had significant impact on the reported BG trends. To the best of our knowledge, this was the first study since the roll out of DTG in Uganda documenting the incidence of T2DM and describing the BG changes in the first year of treatment.

## Conclusion

We demonstrated that the incidence of T2DM in Ugandan ART naïve patients over 48 weeks of DTG anchored ART is low. Nevertheless, there was a trend towards rising blood glucose after week 12. We also demonstrated that longitudinal changes in BG were independent of conventional risk factors of T2DM in the first 48 weeks of therapy. Restrictive use of DTG in Ugandan PLHIV perceived to have a high risk for T2DM may be uncalled for.

## Supplementary Information

**Additional file 1. Table S1**: Sensitivity analysis comparing baseline characteristics of participants who dropped out and those who did not.

## Data Availability

The datasets used and/or analyzed during the current study are available from the corresponding author on reasonable request.

## Abbreviations

ART: Anti-retroviral therapy
NRTI: Nucleoside reverse transcriptase inhibitors
NNRTI: Non-nucleoside reverse transcriptase inhibitors
DTG: Dolutegravir
WHO: World Health Organization
IDI: Infectious Diseases Institute
DKA: Diabetic ketoacidosis
T2DM: Type 2 diabetes mellitus
PLHIV: People living with HIV
OGTT: Oral glucose tolerance test
CDC: Center for diseases control
BMI: Body Mass Index
GPAQ: Global Physical Activity Questionnaire
AUDIT: Alcohol use disorders identification test
FBG: Fasting blood glucose
2hBG: 2-Hour blood glucose
MET: Metabolic equivalent of task
LDL: Low density lipoprotein
HDL: High density lipoprotein
