# Supplementary material for: Blood glucose trajectories and incidence of diabetes mellitus in Ugandan people living with HIV initiated on dolutegravir
Source: AIDS Res Ther. 2023 Mar 13;20:15. doi: 10.1186/s12981-023-00510-6 (PMC10009965; doi:10.1186/s12981-023-00510-6)
Supplement: Supplementary file 1 — Additional file 1. Table S1: Sensitivity analysis comparing baseline characteristics of participants who dropped out and those who did not. [file 12981_2023_510_MOESM1_ESM.docx]

**Blood glucose trajectories and Incidence of pre-diabetes and diabetes mellitus in Ugandan people living with HIV on dolutegravir for 48 weeks.**

Frank Mulindwa^1,2^, Barbara Castelnuovo^1^, Nele Brusselaers^2,3,^ Robert Bollinger^4^, Joshua Rhein^5^, Mutebi Edrisa^6^, Allan Buzibye^1^, Willington Amutuhaire^7^, George Yendewa^7^, Sarah Nabaggala^1^, [Eva Laker Agnes Odongpiny](https://onlinelibrary.wiley.com/action/doSearch?ContribAuthorRaw=Odongpiny%2C+Eva+Laker+Agnes)^1^, Ronald Kiguba^8^, Aisha Nakawooza^1^, Simon Dujanga^6^, Martin Nabwana^9^ and Jean-Marc Schwarz^10,11^

**Affiliations:**

1. *Makerere University Infectious Diseases Institute, Uganda.*
2. *Global Health Institute, Antwerp University, Antwerp, Belgium*
3. *Centre for Translational Microbiome Research, Department of Microbiology, Tumour and Cell Biology, Karolinska University, Sweden.*
4. *Johns Hopkins University, Baltimore, USA*
5. *University of Minnesota School of Medicine, Division of Infectious Diseases, Minnesota, USA*
6. *Makerere University, College of Health Sciences, Internal Medicine department, Kampala, Uganda.*
7. *Case Western Reserve University, Department of Internal Medicine, USA.*
8. *Department of Pharmacology and Therapeutics, College of Health Sciences Makerere University, Kampala, Uganda*
9. *Makerere University Johns Hopkins Collaboration HIV clinic, Kampala, Uganda.*
10. *University of California San Francisco, School of Medicine, San Francisco, USA*
11. *Touro University California College of Osteopathic Medicine, Vallejo, California*

**Corresponding author:**

Dr. Frank Mulindwa

Makerere University Infectious Diseases Institute, Uganda.

Global Health Institute, Antwerp University, Antwerp, Belgium

Email: [fmulindwa@idi.co.ug](mailto:fmulindwa@idi.co.ug)

**Table S1. Sensitivity analysis comparing baseline characteristics of participants who dropped out and those who did not.**

| **Characteristic** | **Did not drop out n=243** | **Dropped out n=63** | **P-value** |
| --- | --- | --- | --- |
| **Age,** Median (IQR) | 31 (27, 38) | 32 (27, 38) | 0.710 |
| **Sex** |  |  |  |
| Female | 140 (57.6) | 41 (66.1) |  |
| Male | 103 (42.4) | 21 (33.9) |  |
| **Baseline CD4 cell count**, Median (IQR) | 318 (163, 524) | 344.5 (183, 627) | 0.729 |
| **Level of education** |  |  |  |
| Uneducated | 3 (1.2) | 0 (0) |  |
| Primary | 129 (53.1) | 33 (54.1) |  |
| Secondary | 99 (40.7) | 27 (44.3) |  |
| Tertiary | 12 (4.9) | 1 (1.6) | 0.733* |
| **Religion** |  |  |  |
| Christian | 188 (77.4) | 55 (88.7) |  |
| Muslim | 55 (22.6) | 7 (11.3) | **0.048** |
| **Residence** |  |  |  |
| Rural | 18 (7.4) | 5 (8.1) |  |
| Urban | 225 (92.6) | 57 (91.9) | 0.792* |
| **Employment** |  |  |  |
| No | 34 (14) | 21 (33.9) |  |
| Yes | 209 (86) | 41 (66.1) | **<0.001*** |
| **Marital status** |  |  |  |
| Single | 135 (55.6) | 42 (67.7) |  |
| Married | 108 (44.4) | 20 (32.3) | 0.083 |
| **Tuberculosis status** |  |  |  |
| No symptoms | 203 (83.5) | 54 (87.1) |  |
| TB suspect | 31 (12.8) | 5 (8.1) |  |
| TB disease | 9 (3.7) | 3 (4.8) | 0.545* |
| **Baseline blood pressure** |  |  |  |
| Normal BP | 175 (72) | 42 (66.7) |  |
| Pre-hypertension | 48 (19.8) | 14 (22.2) |  |
| Hypertension | 20 (8.2) | 7 (11.1) | 0.663 |
| **HIV clinical stage** |  |  |  |
| Stage 1 | 234 (96.3) | 57 (91.9) |  |
| Stage 2 | 3 (1.2) | 2 (3.2) |  |
| Stage 3 | 4 (1.6) | 2 (3.2) |  |
| Stage 4 | 2 (0.8) | 1 (1.6) | 0.265* |
| **Body Mass Index (BMI)** |  |  |  |
| Underweight (<18.5) | 24 (9.9) | 8 (12.7) |  |
| Normal (18.5-24.9) | 148 (60.9) | 38 (60.3) |  |
| Overweight (25.0-29.9) | 57 (23.5) | 10 (15.9) |  |
| Obese (≥30) | 14 (5.8) | 7 (11.1) | 0.273* |
| **Waist circumference** |  |  |  |
| Normal | 160 (65.8) | 40 (64.5) |  |
| Increased risk of cardiometabolic  complications | 45 (18.5) | 12 (19.4) |  |
| Substantially increased risk of  cardiometabolic complications | 38 (15.6) | 10 (16.1) | 0.981 |
| **Smoking status** |  |  |  |
| Smoker | 14 (5.8) | 1 (1.6) |  |
| Non-smoker | 229 (94.2) | 62 (98.4) | 0.322* |
| **Physical activity** |  |  |  |
| GPAQ<600 MET minutes | 46 (18.9) | 20 (32.3) |  |
| GPAQ≥600 MET minutes | 197 (81.1) | 42 (67.7) | **0.023** |
| **Alcohol consumption** |  |  |  |
| No consumption | 139 (57.2) | 43 (68.3) |  |
| Low risk alcohol consumption | 67 (27.6) | 11 (17.5) |  |
| Hazardous alcohol consumption | 19 (7.8) | 5 (7.9) |  |
| Risk of alcohol dependence | 18 (7.4) | 4 (6.3) | 0.374* |
| **Laboratory investigations, Median (IQR)** |  |  |  |
| Creatinine (mg/dl) | 0.83 (0.72, 0.95) | 0.79 (0.7, 0.88) | 0.320 |
| LDL (mg/dl) | 78.11 (59.55, 93.97) | 73.47 (54.91, 102.86) | 0.292 |
| HDL (mg/dl) | 31.71 (25.91, 39.83) | 33.64 (26.68, 38.67) | 0.384 |
| Total cholesterol (mg/dl) | 136.12 (117.94, 158.55) | 134 (115.24, 165.89) | 0.695 |
| Triglycerides (mg/dl) | 90.35 (69.09, 117.8) | 99.2 (74.4, 132.86) | 0.219 |
| Baseline fasting blood glucose, Median (IQR) | 90 (84.6, 95.4) | 88.2 (84.6, 95.4) | 0.577 |
| Baseline 2-hour blood glucose, Median (IQR) | 111.6 (95.4, 126) | 102.6 (88.2, 126) | **0.026** |

** fisher’s exact p-value was reported*
